# MCL and Mincle: C-Type Lectin Receptors That Sense Damaged Self and Pathogen-Associated Molecular Patterns

**DOI:** 10.3389/fimmu.2014.00288

**Published:** 2014-06-23

**Authors:** Mark B. Richardson, Spencer J. Williams

**Affiliations:** ^1^School of Chemistry and Bio21 Molecular Science and Biotechnology Institute, University of Melbourne, Parkville, VIC, Australia

**Keywords:** cord factor, C-type lectin receptors, glycolipids, PAMP, DAMP, T cell

## Abstract

Macrophage C-type lectin (MCL) and macrophage inducible C-type lectin (Mincle) comprise part of an extensive repertoire of pattern recognition receptors with the ability to sense damage-associated and pathogen-associated molecular patterns. In this review, we cover the discovery and molecular characterization of these C-type lectin receptors, and highlight recent advances in the understanding of their roles in orchestrating the response of the immune system to bacterial and fungal infection, and damaged self. We also discuss the identification and structure–activity relationships of activating ligands, particularly trehalose dimycolate and related mycobacterial glycolipids, which have significant potential in the development of T_H_1/T_H_17 vaccination strategies.

## Introduction

**Figure 1:**
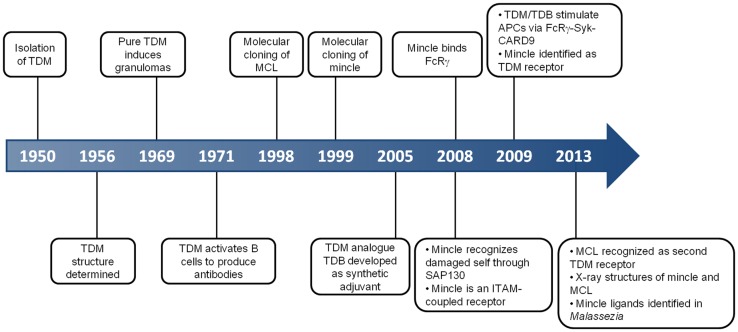
**Key events in the history of MCL and Mincle**. This timeline depicts some of the critical discoveries that have shaped our understanding of the function and roles of MCL and Mincle.

Macrophage C-type lectin (MCL; Clec4d, ClecSf8) and macrophage inducible C-type lectin (Mincle; Clec4e, ClecSf9) are transmembrane germline-encoded pattern recognition receptors (PRRs) that form part of the innate immune system. These C-type lectin receptors (CLRs) recognize damage-associated molecular patterns (DAMPs) and enable immune sensing of damaged self, and pathogen-associated molecular patterns (PAMPs) from a growing list of bacteria and fungi (Figure 1) (1, 2). The PAMPs notably include mycobacterial trehalose dimycolate (TDM, cord factor), and appear to play a significant roles in the immune response to certain bacterial and fungal infections. In the case of Mincle, recognition of PAMPs is mediated through the carbohydrate binding part of the carbohydrate recognition domain (CRD) in the extracellular region of the CLR, whereas recognition of DAMPs occurs through a distinct region of the CRD (3). For both CLRs, signal transduction occurs through the immunoreceptor tyrosine-based activation motif (ITAM)-containing adaptor molecule Fc receptor γ-chain (FcRγ). Ligand binding to Mincle leads to phosphorylation of the ITAM of FcRγ and recruitment of spleen tyrosine kinase (Syk) (3). Syk recruitment by FcRγ leads to nuclear factor kappa-light-chain-enhancer of activated B cells (NF-κB) activation through Card9–Bcl10–MALT1 signalosomes, pivotal regulators that link innate and adaptive immune responses (4). DNA transcription leads to the production of cytokines and chemokines that shape the development of naïve T cells into effector T helper (T_H_) cell T_H_1 and T_H_17 subtypes (5). There is a growing interest in the immunological roles of MCL and Mincle for the development of defined synthetic adjuvants for T_H_1/T_H_17 vaccination, and due to the involvement of these CLRs in the recognition of bacterial and fungal pathogens, and the response to dysregulated cell death.

## MCL and Mincle: C-Type Lectin Receptors

**Figure 2:**
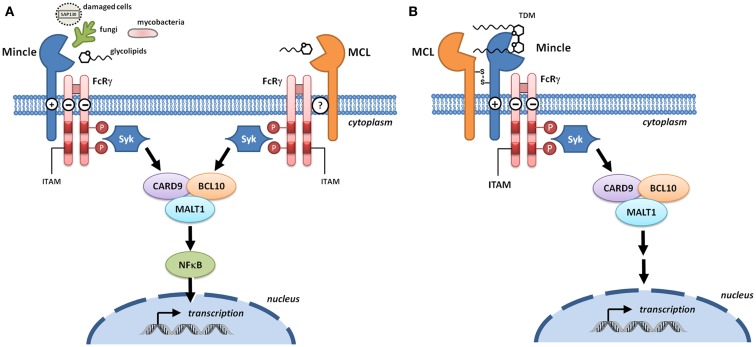
**Signaling through Mincle and MCL**. **(A)** Mincle and MCL pair with the signaling adaptor molecule Fc receptor γ-chain (FcRγ). With Mincle this association is driven by the presence of a positively charged arginine in the transmembrane region whereas the corresponding residue is not present in MCL. Upon binding to DAMPs or fungal and bacterial PAMPs, phosphorylation of the immunoreceptor tyrosine-based activation motifs (ITAMs) of FcRγ recruits spleen tyrosine kinase (Syk) and induces signaling through the Card9–Bcl10–MALT1 complex. Transcription results in the formation of immunoregulatory cytokines and chemokines. **(B)** Heterodimers of MCL and Mincle have the capacity to sense trehalose dimycolate (TDM). In these complexes, Mincle recognizes the carbohydrate headgroup while MCL recognizes the lipid tail, and Mincle acts as a bridge to enable formation of a functional MCL–Mincle–FcRγ complex.

The genetic and molecular analysis of MCL and Mincle preceded the understanding of their function. MCL was originally cloned as a mouse macrophage-restricted C-type lectin (6). Mincle was cloned as a transcriptional target of the nuclear factor NF-IL-6, which binds the interleukin-1 (IL-1) responsive element of the IL-6 gene (7). Mincle RNA was induced upon exposure to inflammatory stimuli including lipopolysaccharide (LPS), tumor necrosis factor-α (TNFα), IL-6, and interferon-γ (IFNγ). Cloning/identification of the human and rat genes followed shortly thereafter (8, 9). According to the HUGO Gene Nomenclature Committee, the gene encoding MCL is CLEC4D (formerly CLECSF8) and that encoding Mincle is CLEC4E (formerly CLECSF9). Both CLRs are located on mouse chromosome 6 and human chromosome 12, clustered with closely related CLRs of the Dectin-2 family (Dectin-2, DCIR, DCAR, and BDCA-2) within the telomeric region of the natural killer complex (10). Sequence analyses predict type 2 transmembrane proteins with an N-terminal cytoplasmic tail, a transmembrane region, a membrane proximal stalk, followed by a single C-terminal C-type lectin domain (11). The transmembrane region of Mincle contains a positively charged arginine that mediates interaction with ITAM-bearing adaptors; a direct interaction of Mincle with FcRγ was obtained by immunoprecipitation of Mincle using an anti-FcRγ antibody in transfected human embryonic kidney cells, with the precipitation abolished upon substitution of arginine by leucine (Figure 2A) (3). This interaction is critical for the ability of Mincle to signal as Mincle-dependent signaling is lost in the leucine mutant (3).

Macrophage C-type lectin is an FcRγ coupled receptor (12) that signals through Syk (13); however the nature of the adaptor protein interaction is unclear (Figure 2A). MCL does not contain an arginine residue within the transmembrane domain that is typically required for association with FcRγ and so a direct interaction with this adaptor seems unlikely. Immunoprecipitation of MCL in a rat myeloid cell line led to co-precipitation with FcRγ (14) but this could not be replicated in a transfected non-myeloid cell line, indicating that additional cellular components are required for this interaction (15). Co-transfection of non-myeloid cells with MCL, Mincle, and FcRγ followed by immunoprecipitation gave evidence for a covalently linked heterodimer of MCL and Mincle that associated with FcRγ, suggesting that Mincle acts as a bridge for the interaction between MCL and FcRγ (Figure 2B) (15, 16). Phagocytic internalization of anti-Mincle and anti-MCL coated beads provided strong evidence for a functional complex of MCL–Mincle–FcRγ on the cell surface. In primary rat peritoneal macrophages, expression of Mincle and MCL is tightly coupled, suggesting that Mincle–MCL heterodimers are formed. It was proposed that in Mincle–MCL heterodimers, TDM binding is mediated through carbohydrate binding to Mincle and lipid binding to MCL, a model that explains the ability of MCL to recognize soluble TDM but not TDM associated with mycobacterial cells.

In humans and rodents, Mincle is expressed on monocytes, macrophages, neutrophils, dendritic cells (DCs), and some subsets of B cells (3, 7, 17–19), and has been additionally detected on T cells and concanavalin A blasts in rats (9). Mincle is inducible on mouse macrophages upon activation of TLR4 (18), and is also inducible on human B cells through activation of TLR9 (17). In guinea pig, Mincle is expressed in the spleen, lymph nodes, and peritoneal macrophages and is up-regulated upon stimulation by zymosan and TDM (20).

Macrophage C-type lectin expression was first characterized as macrophage-restricted in mice (6). Human MCL is expressed on macrophages (synovial, peritoneal, and blood monocyte-derived) and Langerhans cells (8), and on neutrophils, monocytes, and immature and mature DCs (13). In rats, expression of MCL has been detected on macrophages, neutrophils, DCs, B cells, and T cells (9, 14). Upon treatment with various stimuli including PAM3CSK4, TNF-α, and IFNγ, MCL expression is inducible on monocytes and neutrophils (8, 13), but considerable variation is found between studies. For example, using monocytes from human donors, Arce et al. qualitatively demonstrated that IL-6, TNF-α, IL-10, and IFNγ caused up-regulation of MCL expression, whereas LPS caused down-regulation (8). In contrast, using similar protocols and cell lines, Graham et al. obtained different results (13). LPS caused up-regulation; and TNF-α, IFNγ, IL-6, and IL-10 did not change expression levels, although a less than twofold change in expression levels was observed. Expression of MCL on rat macrophages is inducible with IFNγ and pro-inflammatory stimuli from Gram-negative bacteria (14).

Carbohydrate recognition by lectins is frequently associated with conserved glutamic acid–proline–asparagine (EPN) or glutamine–proline–aspartate (QPD) motifs within the CRDs of C-type lectins (see Box 1) (21). Mincle contains an EPN motif within its CRD, leading to the suggestion of mannose/fucose/*N-*acetylglucosamine/glucose specificity, which is commonly seen for such motifs. While MCL contains a conserved Ca^2+^ binding site, it lacks a canonical EPN or QPD motif, instead possessing an EPX motif in rat (X = K) and human (X = D), whereas mouse possesses an ESN sequence. Insight into carbohydrate recognition by Mincle and MCL was assessed for six hexoses: mannose, fucose, *N-*acetylglucosamine, glucose, galactose, and *N-*acetylgalactosamine (22). Relative affinities for Mincle were mannose ~ fucose > glucose > *N-*acetylglucosamine > galactose ~ *N-*acetylgalactosamine. These relative affinities were roughly paralleled by MCL: mannose ~ fucose > glucose ~ *N-*acetylglucosamine > galactose ~ *N-*acetylgalactosamine, although this CLR bound only very weakly to all hexoses examined. A screen of a 326-member carbohydrate microarray for ligands for a soluble MCL–Fc fusion protein failed to identify any carbohydrate ligands for this receptor (13), suggesting that MCL may not in fact be a carbohydrate binding lectin.

Box 1 **C-type lectins: structure and classification.**

C-type lectins are a subclass of lectins that are distinguished by a requirement for Ca^2+^ for binding (21). Crystallographic studies reveal that C-type lectins contain a compact globular structure that comprises the carbohydrate recognition domain (CRD). The CRD contains conserved amino acid residue motifs, and which allows the prediction of new C-type lectins on the basis of sequence data. Somewhat confusingly, it has since been found that many predicted C-type lectin CRDs do not bind either carbohydrates or Ca^2+^. C-type lectins can be soluble or transmembrane proteins. An early classification of C-type lectins was introduced on the basis of (1) the identification of mannose- or galactose-specific binding motifs [glutamic acid–proline–asparagine (EPN) or glutamine–proline–aspartate (QPD) motifs, respectively] in well-characterized mannose- and galactose-specific lectins known at the time, and (2) conversion of the ligand specificity from mannose to galactose by mutagenesis (23). Although based on compelling studies at the time, this further adds to the confusion as some predicted mannose or galactose binding C-type lectins do not in fact bind to these carbohydrates. C-type lectins are classified into 17 groups; Mincle and MCL fall into Group II of the CTL family and are typically grouped in with a subset of CTLRs termed the Dectin-2 cluster that contains other PRRs including Dectin-2, DCIR, DCAR, and BDCA-2 (10).

Functional studies of CLRs have been greatly accelerated through the development of reporter cell lines. In the case of Mincle, a useful reporter is a T cell hybridoma that expresses Mincle and FcRγ, as well as green fluorescent protein (GFP) under the control of the transcription factor nuclear factor of activated T cells (NFAT) to detect ITAM-mediated signals (3). An MCL reporter strain in which LacZ β-galactosidase is expressed under the control of NFAT in a T cell hybrid has been reported (15).

## Mincle in the Damaged Cell Response

Dead cells activate Mincle-expressing cells. The factor causing activation was identified to be spliceosome-associated protein 130 (SAP130) (3). SAP130 binds to Mincle in a Ca^2+^ independent manner and mutation of the EPN motif of Mincle did not affect binding. Conversely, a mutant in the region recognized by a blocking antibody, VEGQW, was not activated by dead cells, suggesting that SAP130 binds to the CRD but at a distinct site to that of carbohydrate binding. SAP130 derived from either living or dead cells has a similar ability to activate through Mincle. As SAP130 is located in the nucleus in live cells, Mincle recognition of dead cells must occur after translocation to the external milieu. SAP130 therefore acts as a DAMP, providing an alarm signal for dysregulated cell death. Gamma-ray irradiation causes cell death in the thymus and induces neutrophil infiltration. Mincle RNA is up-regulated after irradiation, but use of a Mincle-blocking antibody suppressed neutrophil infiltration into the thymus after irradiation. The observation that macrophage inflammatory protein 2 (MIP-2), a specific signal produced by thymic macrophages, was inhibited by the Mincle-blocking antibody suggests that Mincle activation by ligands induces the production of inflammatory cytokines and/or chemokines.

Ischemia results in dysregulated cell death and the exit of cellular components, suggesting the possible involvement of Mincle-mediated inflammation. Mincle knockout mice show a better outcome after stroke (24). Cerebral ischemia results in induction of Mincle expression in immune, neuronal, and endothelial cells, which paralleled increases in SAP130 expression (25). Levels of phosphorylated-Syk (p-Syk) were raised following ischemia suggesting that Mincle activation leads to increased levels of p-Syk. Application of the Syk inhibitor piceatannol reduced infarct volume and swelling, suggesting that signaling through the Syk-Card9–Bcl10–Malt1 axis is an important factor in the response to ischemia.

## Identification of Mycobacterial Glycolipids as MCL and Mincle Antigens and Their Role in Mycobacterial Infection

Research in the areas of mycobacterial immunogenicity and C-type lectins began to merge with the report that TDM activates macrophages and DCs via Syk–Card9–Bcl10–Malt1 signaling to produce innate activation that was distinct from that produced by Toll-like receptor ligands (4). Activation of antigen presenting cells was independent of Dectin-1 but required the ITAM-bearing signaling adaptor FcRγ, leading to the suggestion that a range of C-type lectins, including Mincle, were possible TDM receptors (4). Independently, Mincle had been shown to be associated with FcRγ (3). Two contemporaneous reports identified Mincle as a TDM receptor. Using Mincle-expressing reporter cells, Yamasaki and co-workers showed that while heat-killed mycobacteria could activate Mincle-expressing cells, delipidated cells could not, and the activity was located within the lipid extract (26, 27). Sub-fractionation of this extract identified TDM as the activating species. Independently, Lang and co-workers mined a gene expression array database for genes expressed and up-regulated in bone marrow macrophages treated with TDB (28). The candidate Mincle was expressed as an Fc fusion protein and was shown by ELISA to bind to TDB and TDM in a dose-dependent manner. TDM is an important glycolipid produced by all mycobacteria that possesses potent immunostimulatory properties, in particular the ability to cause granulomas. Using Mincle^−/−^ mice, Mincle was shown to be essential for the granulomatous response to TDM, providing compelling evidence that Mincle is a major TDM receptor (26). While initial reports demonstrated that TDM activates mouse Mincle, a recent report using a reporter cell has shown that TDM also activates human Mincle (29).

The discovery that MCL is a receptor for TDM arose from the initial observation that MCL is expressed on neutrophils and monocytes and triggers cellular activation through Syk (13). The observation that resting macrophages barely express Mincle, yet addition of TDM drives Mincle expression, suggested the existence of another TDM receptor (12). Innate immune responses were impaired in MCL-deficient mice, including the TDM-induced acquired immune response, experimental autoimmune encephalomyelitis (EAE) (12). Further, MCL was shown to be required to drive Mincle expression in a Clec4e–GFP fusion reporter mouse (12). Daws reported that MCL reporter strains were not responsive to intact mycobacteria and argued that this observation is consistent with MCL recognizing the lipid portion of TDM, which is exposed in the purified antigen but embedded in the bacterial cell wall in intact bacteria (15). Recent studies of guinea pig homologs of Mincle (gpMincle) and MCL (gpMCL) revealed that only gpMincle binds TDM and that gpMincle is constitutively expressed (20). gpMCL lacks the hydrophobic region proposed to be involved in TDM binding, although it does bind FcRγ. This work suggested that of these two receptors, only gpMincle is involved in guinea pig immune responses against mycobacteria and that the functions of MCL and Mincle in recognition of mycobacteria are not conserved between humans and guinea pig.

The identification of Mincle and MCL as TDM receptors and establishing that Mincle is required for the TDM-induced granulomatous response has resolved longstanding questions in the field. However, the connection of Mincle and MCL to the anti-mycobacterial response in the context of infection has been less clearly answered (30). An early role for the cytosolic adaptor caspase recruitment domain family, member 9 (Card9) in pulmonary tuberculosis was demonstrated when Card9^−/−^ mice were shown to succumb rapidly to aerosol infection by *Mycobacterium tuberculosis* H37Rv (31). Comparison of bone marrow derived macrophages of wild-type and Card9^−/−^ mice when infected with *M. tuberculosis* revealed a significant reduction in the homozygous mutant of the pro-inflammatory cytokines, TNF, IL-1β, and IL-6, and reduced IL-12 and CCL5, compared to wild-type. Heat-killed *Mycobacterium bovis, Mycobacterium smegmatis*, and *M. tuberculosis* H37Rv all activated an NFAT-driven GFP reporter strain that expresses Mincle and FcRγ, with activation ablated upon mutation of the Mincle CRD EPN motif to QPD (26). The effect of Mincle deletion upon *M. tuberculosis* infection has been studied in Mincle^−/−^ mice. While in the absence of Mincle, macrophages did not produce the reactive nitrogen species NO_2_^−^ upon TDM stimulation, Mincle-deficient macrophages were responsive to *M. tuberculosis* infection, and co-stimulation with IFNγ resulted in normal levels of granulocyte colony-stimulating factor (G-CSF), TNF and NO_2_^−^. Mincle^−/−^ mice did not form granulomas upon stimulation with TDM, the same mice forms granulomas that were indistinguishable from wild-type upon infection with *M. tuberculosis*. Furthermore, while TDM resulted in induction of T_H_1 and T_H_17 cells, Mincle^−/−^ mice infected with *M. tuberculosis* developed normal T_H_1 and T_H_17 immune responses, indicating that in the context of *M. tuberculosis* infection, Mincle is not required for instructing maturation of naïve T cells. This observation is consistent with studies with *Fonsecaea pedrosoi* that suggests that while specific signals are transmitted by Mincle during vaccination with purified TDM, PRR co-stimulation during infection leads to a cocktail of cytokines and chemokines that shape T cell development (32) (see Box 2).

Box 2 **The role of MCL and Mincle in T cell development.**

**Figure 3:**
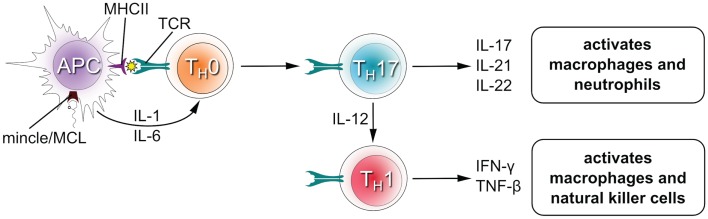
**T cell differentiation upon stimulation of Mincle/MCL**.

Upon recognition of their cognate antigen, naïve CD4^+^ T cells (T_H_0 cells) can differentiate into novel effector CD4^+^ T cell lineages that can regulate or assist active immune responses (Figure 3). The fate of the transforming T_H_0 cell is determined by the pattern of cytokines it receives at the moment of antigen recognition by the T cell receptor. Activation of Mincle and MCL induces expression of interleukins-1 (IL-1) and IL-6 in antigen presenting cells (4, 19, 26, 33, 34), which flood the microenvironment of the transforming T_H_0 cell and shape the development of T_H_1 and T_H_17 phenotypes in both humans and mice (12, 28). The T_H_17 lineage is defined by the production of IL-17, which induces and mediates pro-inflammatory responses leading to the recruitment of monocytes and neutrophils, which can clear infections (35). T_H_1 cells characteristically produce IFNγ, an activator of natural killer cells (which provide direct killing of pathogens) and macrophages (leading to phagocytosis).

MCL-deficient mice have defective immune responses to mycobacterial infection. Induction of TNF and MIP-2 RNA was impaired in bone marrow derived macrophages from MCL^−/−^ mice (12). Further, MCL^−/−^ mice gave impaired IFNγ levels in Mantoux test responses when stimulated with purified protein derivative compared to wild-type.

Glycerol monomycolate (GroMM) has been identified as a Mincle-activating lipid (29). Using NFAT–GFP reporter strains, it was shown that human Mincle-expressing cells could be activated by GroMM, although less potently than for TDM. While mouse Mincle-expressing cells were not activated by GroMM, transgenic mice expressing human Mincle gained the ability to recognize GroMM, and injection of GroMM liposomes into the skin of these mice resulted in infiltration of macrophages and eosinophils. In primary human monocyte-derived macrophages, GroMM produced TNFα in response to GroMM that could be blocked by an anti-human Mincle antibody, demonstrating that this glycolipid is a ligand for human, but not mouse Mincle.

## Structure–Activity Relationships of Trehalose and Glycerol-Based Antigens for Mincle

**Figure 4:**
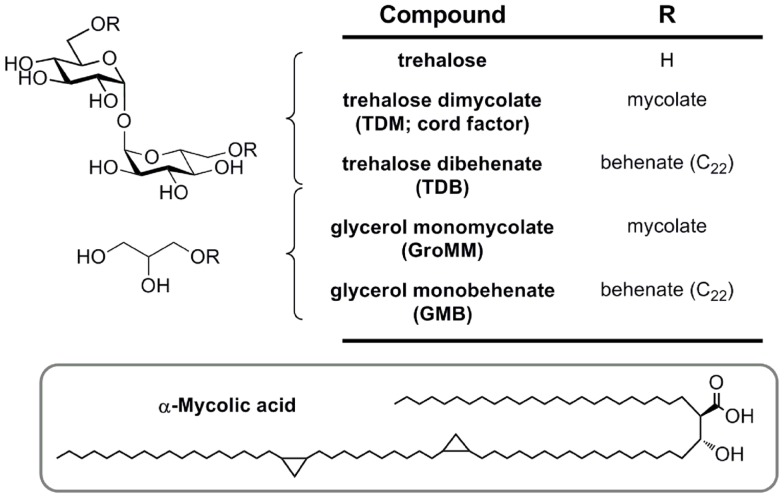
**Mycobacterial mycolic acid-based lipids and synthetic analogs (TDB and GMB), structurally defined glycolipid ligands for MCL and Mincle**.

Mincle is potently activated by TDM (Figure 4). Treatment of TDM with trehalase, which apparently cleaves this molecule into glucose monomycolate (GMM), abolished Mincle binding (26). This result suggests that Mincle specifically recognizes the two glucose residues within TDM and is particularly interesting as GMM is a glycolipid produced upon infection by *M. tuberculosis* and which itself is a potent antigen when presented to T cells by CD1b (36). Cells are activated to similar degrees by the TDM analog trehalose dibehenate, which suggests that complex mycolate structures are not necessary for Mincle activation. Measurement of the direct interaction of Mincle with TDM analogs is limited by their poor solubility and so accurate data has only been obtained with shorter acyl groups. For bovine Mincle, affinities of trehalose diesters generally increase with increasing chain length (37). Trehalose monoesters are also effective ligands for human and bovine Mincle with affinities increasing up to 6-*O*-lauryltrehalose (C_12_) and 6-*O*-octanoyltrehalose, respectively (37, 38). Trehalose itself is a weak ligand for Mincle, and methyl α-glucoside, representing a monomer of trehalose, possesses 36-fold weaker affinity for Mincle (37). The C_22_ and C_26_ trehalose monoesters were able to activate mouse macrophages in a Mincle-dependent manner (34).

Glycerol monomycolate is an antigenic ligand for human, but not mouse Mincle (Figure 4) (29). Plate-bound GroMM is less potent than TDM in an NFAT–GFP reporter cell assay. Glycerol monobehenate (GMB) possesses similar activity to GroMM toward human Mincle and gave only marginal responses for mouse Mincle reporter cells.

## Structures of CRDs of MCL and Mincle

The classification of Mincle and MCL as CLRs leads to the prediction that the CRD domain will conform to the typical domains seen for this class of proteins. Two recent papers have independently reported structures of Mincle and MCL C-type lectin domains. Maenaka and co-workers reported the structures of human Mincle and MCL, recombinantly expressed in *Escherichia coli* (38). While wild-type human Mincle failed to crystallize, the I99K mutant (which matches the equivalent residue in human MCL) provided diffracting crystals. Drickamer and co-workers have reported the structure of bovine Mincle, expressed using a similar approach and crystallized without the need for mutagenesis (37).

**Figure 5:**
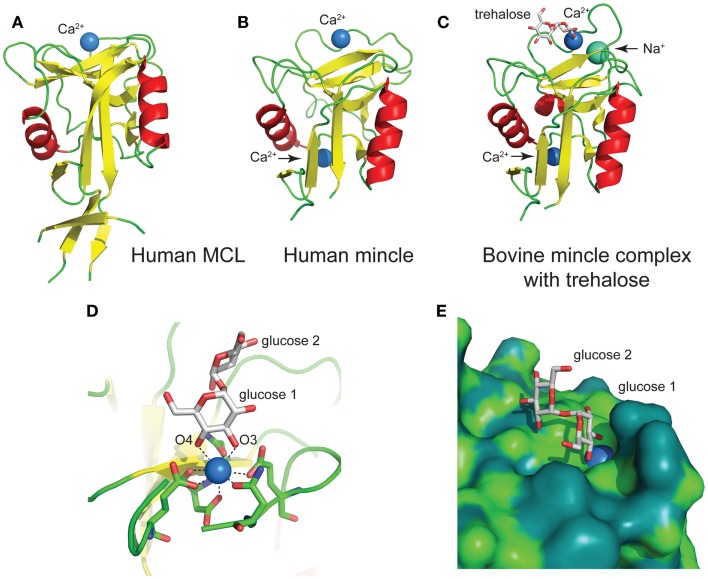
**Structures of the C-type lectin domains of (A) human MCL (PDB: 3whd); (B) human Mincle (PDB: 3wh3); (C) bovine Mincle complex with trehalose (PDB: 4kzv); (D) interactions with Ca^2+^ at the primary glucose binding site of bovine Mincle; and (E) putative hydrophobic channel adjacent to the primary glucose binding site of bovine Mincle complex with trehalose (the protein surface is colored according to the underlying atoms: light green, nitrogen/oxygen; dark green, carbon)**.

The human MCL C-type lectin domain comprises a globular fold containing two alpha helices around a beta-strand core, with a single Ca^2+^ bound (Figure 5A) (38). The human and bovine Mincle C-type lectin domains reveal largely identical folds, and both contain two Ca^2+^ ions, with the bovine structure containing an additional Na^+^ ion (Figure 5B) (38). Structural insight into carbohydrate recognition by Mincle was obtained through a complex of bovine Mincle with trehalose (Figure 5C) (37). In this complex, only one pyranose ring of trehalose binds to calcium through O3 and O4 of glucose (Figure 5D). Insight into a possible molecular basis for signal transduction was obtained through a conformational change in a loop between Asn-170 and Asp-177 near the conserved Ca^2+^ upon biding of trehalose. Examination of the surface of Mincle near the trehalose binding site identified a hydrophobic channel lined with lipophilic amino acids that is adjacent to the 6-OH of the Ca^2+^-bound glucose ring, which was proposed to comprise a lipid binding channel (Figure 5E).

## MCL and Mincle in Fungal Disease

Effective protection of host from pathogenic fungi and clearance of infection requires a coordinated immune response from both T_H_1 and T_H_17 cells that restrict fungal cell growth and drive phagocytic clearance (2, 5). The Syk–Card9–Bcl10–MALT1 signaling axis leads to NF-κB activation, which drives the expression of multiple cytokines including IL-1, IL-6, and IL-23 that facilitate T_H_17 cell differentiation, and IL-12, which is essential for T_H_1 differentiation (see Box 2).

Yamasaki and co-workers screened 50 species of pathogenic fungi as Mincle activators using a NFAT–GFP reporter cell line (39). Of these only *Malassezia* species, including *Malassezia pachydermatis, Malassezia dermatis, Malassezia japonica, Malassezia nana, Malassezia slooffiae, Malassezia sympodialis*, and *Malassezia furfur*, induced strong NFAT–GFP activation. Interestingly, particularly given results from other laboratories, *Candida albicans* did not result in NFAT–GFP expression. In normal skin, *Malassezia* spp. are commensals, however in atopic/eczema and psoriasis, these fungi can elicit inflammatory responses in skin lesions and can cause diseases such as tinea versicolor, atopic dermatitis, and lethal sepsis. Reporter cells with mutant Mincle mutated in the CRD were not activated by *Malassezia* indicating that the likely ligand is a carbohydrate, and as discussed below, subsequent work led to the isolation of glycolipids including gentiobiosyl diacylglycerides and a complex mannosyloxylstearyl mannitol glycolipid (40). Co-culture of *M. pachydermatis* with wild-type murine bone marrow macrophages resulted in production of MIP-2, TNFα, keratinocyte-derived chemokine (CXCL1), and IL-10 cytokines, which were reduced but not ablated in Mincle^−/−^ macrophages. When *M. pachydermatis* was injected into the peritoneal cavity, wild-type mice produced IL-6 and TNF, which was reduced in the Mincle^−/−^ mutant, suggesting that Mincle is important in immune responses to these fungi.

Chromoblastomycosis is a chronic skin infection caused by fungi including *F. pedrosoi*. *F. pedrosoi* is recognized by Mincle, resulting in production of high levels of IL-10 and low levels of TNF and IFNγ (32). Under these conditions, *F. pedrosoi* establishes a chronic infection which is unable to be cleared. Co-stimulation of macrophages and DCs infected with *F. pedrosoi* with the TLR2 agonist PAM3CSK4, the TLR7 agonist Imiquimod, or the TLR4 agonist LPS gave robust levels of TNF, suggesting that this fungus fails to cause co-stimulation of PRRs. Indeed, Mincle^−/−^ bone marrow DCs lacked the ability to be co-stimulated by LPS and *F. pedrosoi*, and *F. pedrosoi* infected mice effectively cleared disseminated infections with this fungus when treated with a single dose of LPS. As well, topical application of Imiquimod significantly decreased fungal burden in skin after subcutaneous infection. To gain further insight into the mechanisms IL-6 of inflammatory responses, Wevers and co-workers studied stimulation of human DCs with the related fungus *Fonsecaea monophora*, also a causative agent of chromoblastomycosis (41, 42). *F. monophora* triggered the maturation of DCs and production of IL-6, IL-1β, and IL-23, but not IL-12p70. In this case, *F. monophora* is recognized by both Dectin-1 and Mincle, and while activation through Dectin-1 resulted in cytokine production, Mincle activation resulted in suppression of IL-12p70 through suppression of IL-12p35 transcription. This in turn was achieved by Mincle signals targeting the proteasomal degradation of nuclear IRF1 via the ubiquitin E3 ligase Mdm2. Mdm2 activation and translocation was a result of Mincle triggering of the signaling kinase PKB, which was not triggered by Dectin-1 or TLR4. Overall, this process leads to impairment of IL-12 and is important for shaping the development of CD4^+^ T cells toward T_H_17 cells. This appears to be a general phenomenon for Mincle activation by other fungi including *Fonsecaea compacta* and *Cladophialophora carrionii*, and indeed by the TDM mimic TDB. Naïve CD4^+^ T cells co-cultured with DCs primed with the TLR4 agonist LPS differentiate to T_H_1 polarized cells, however in the presence of *F. pedrosoi* and other fungi, these are skewed to a T_H_2 response.

Mincle appears to play crucial roles in *C. albicans* infection. Mincle-deficient mice are more susceptible to systemic candidiasis, and production of TNF-α by macrophages was reduced *in vivo* and *in vitro* (43). The soluble CRD of human and mouse Mincle was found to bind whole *C. albicans* cells (44). However, a subsequent screening study of different *C. albicans* strains did not activate a Mincle-expressing NFAT–GFP reporter strain, leading to the suggestion that strain-specific features are required for Mincle activation (39). The identity of the *C. albicans* ligands is not known.

A direct role for MCL in fungal disease is yet to be demonstrated. No significant differences were observed in the ability of MCL-deficient mice to resist infection with *C. albicans* (13).

## Identification of Mincle Antigens from Fungi

**Figure 6:**
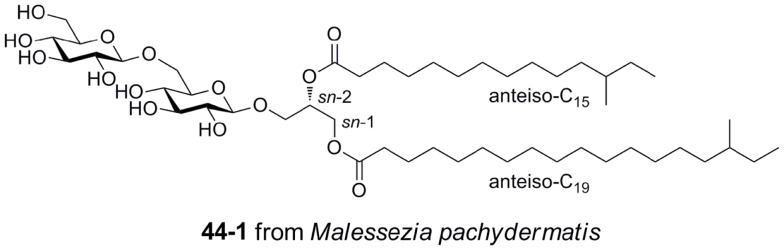
**Gentiobiosyl diacylglycerides from the fungus *Malessezia pachydermatis*, ligands for mouse Mincle**.

Gentiobiosyl diacylglycerides from *M. pachydermatis* have been identified as mouse Mincle ligands (Figure 6) (40). These glycolipids bear anteiso fatty acyl groups (anteiso-C_15_, C_17_, C_19_, or C_20_) at the *sn*-1 and *sn*-2 positions of the glycerol. Four lipoforms were identified with the following substituent permutations (*sn*-1/*sn*-2): anteiso-C_19_/anteiso-C_15_, anteiso-C_17_/anteiso-C_15_, anteiso-C_20_/anteiso-C_15_, and anteiso-C_19_/anteiso-C_17_. All isomers activated Mincle to similar degrees, but were significantly less potent than TDM. While these gentiobiosyl diacylglycerides bear significant resemblance to the glycolipid membrane anchor of lipoteichoic acid, it did not activate via Mincle.

**Figure 7:**
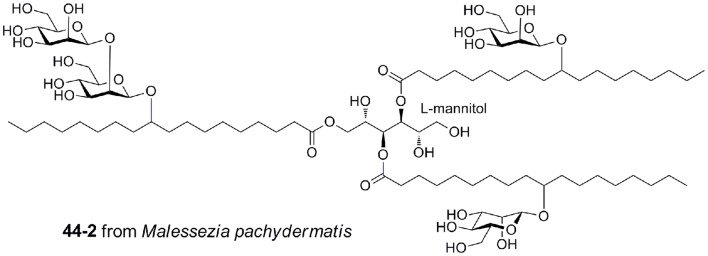
**Mannosyloxylstearyl mannitol glycolipid from *Malessezia pachydermatis*, a ligand for mouse Mincle**.

*Malassezia pachydermatis* produces a complex mannosyloxylstearyl mannitol glycolipid that is a potent activator via mouse Mincle, with a potency approaching that of TDM (Figure 7) (40). This glycolipid comprises β-linked mannose residues attached to 10-hydroxystearic acid and esterified onto an l-mannitol core.

## Conclusion

T cell mediated immune responses induced by PRRs are important in recognition of damaged self and pathogens. The identification of DAMPs and PAMPs that activate Mincle has elevated the importance of this CLR, and the involvement of MCL as an auxiliary CLR that forms heterodimers with Mincle provides the potential for an expansion in the range of ligands recognized by Mincle. MCL and Mincle appear to be an important part of a larger repertoire of PRRs and recent studies of the interplay of Mincle with Dectin-1 and TLRs demonstrate the potential for modulation of immune signals through PRR co-stimulation. The availability of three dimensional X-ray structures of the CRDs of these receptors has unveiled a molecular picture of ligand recognition that may inform development of novel T_H_1/T_H_17 vaccines. However, at this stage it is not clear how Mincle can recognize a diverse range of structurally dissimilar antigens, or the structural changes that lead to signal transduction and transcription. Further, it is likely that additional pathogens that activate MCL and Mincle remain to be identified as well as new ligands from existing pathogens such as *Klebsiella pneumonia* (45) and *F. pedrosoi* that activate through Mincle. It is noteworthy that the majority of studies with MCL and Mincle has been performed on mice and it will require additional work to translate these findings to the human system. The species differences noted in the expression and functional properties of MCL and Mincle, and the selective activation of human, but not mouse Mincle by GroMM, suggest that the development of humanized animal models and cell lines will be essential for understanding the role of these CLRs in human health and disease.

## Conflict of Interest Statement

The authors declare that the research was conducted in the absence of any commercial or financial relationships that could be construed as a potential conflict of interest.
